# DiffNILM: A Novel Framework for Non-Intrusive Load Monitoring Based on the Conditional Diffusion Model

**DOI:** 10.3390/s23073540

**Published:** 2023-03-28

**Authors:** Ruichen Sun, Kun Dong, Jianfeng Zhao

**Affiliations:** School of Electrical Engineering, Southeast University, Nanjing 210096, Chinajianfeng_zhao@seu.edu.cn (J.Z.)

**Keywords:** NILM, non-intrusive load monitoring, diffusion models, deep learning

## Abstract

Non-intrusive Load Monitoring (NILM) is a critical technology that enables detailed analysis of household energy consumption without requiring individual metering of every appliance, and has the capability to provide valuable insights into energy usage behavior, facilitate energy conservation, and optimize load management. Currently, deep learning models have been widely adopted as state-of-the-art approaches for NILM. In this study, we introduce DiffNILM, a novel energy disaggregation framework that utilizes diffusion probabilistic models to distinguish power consumption patterns of individual appliances from aggregated power. Starting from a random Gaussian noise, the target waveform is iteratively reconstructed via a sampler conditioned on the total active power and encoded temporal features. The proposed method is evaluated on two public datasets, REDD and UKDALE. The results demonstrated that DiffNILM outperforms baseline models on several key metrics on both datasets and shows a remarkable ability to effectively recreate complex load signatures. The study highlights the potential of diffusion models to advance the field of NILM and presents a promising approach for future energy disaggregation research.

## 1. Introduction

In recent years, the demand for fine-grained power data has increased, leading to a growing interest in the energy disaggregation technique for obtaining information on appliance-level power consumption. A commonly cited application of this technique is to generate detailed electricity bills, which encourage energy conservation among residents. Additionally, electric power companies can utilize disaggregated power consumption data to calculate Demand Side Response (DSR) resources and evaluate DSR capability. An intuitive way to obtain such data is through Intrusive Load Monitoring (ILM), which involves the direct installation of sensors on target appliances. While ILM yields accurate results, it is generally considered unfeasible for large-scale deployment due to its high cost. On the other hand, Non-Intrusive Load Monitoring (NILM) has gained better application prospects from an economic standpoint. NILM can be viewed as a software sensor that identifies the operating states of individual appliances and estimates their power consumption using only power or current data recorded by the mains meter; thereby, reducing the overall cost.

The practical implementation of NILM has been facilitated by the development of big data technology in the energy industry. Advanced Metering Facilities (AMIs) provide real-time load monitoring data, and modern Artificial Intelligence (AI) algorithms can effectively process massive amounts of data.

In our study, energy disaggregaion is framed as a generation task and a highly promising deep generative model, the diffusion model [1], is employed to reconstruct target power profiles. In the last two years, diffusion models have been gaining significant popularity and have nearly replaced Generative Adversarial Network (GAN) and other generative models due to their ease of training, improved tractability, and flexibility. Diffusion models have demonstrated exceptional performance in various fields, including image generation [2], image segmentation [3], audio synthesis [4] and point cloud reconstruction [5], etc. However, to the best of our knowledge, no published research has investigated the use of diffusion models for NILM. Therefore, this paper proposes DiffNILM, a diffusion probabilistic model for energy disaggregation. The main contributions of our work are as follows:

- DiffNILM is the first NILM framework adopting the diffusion model. Specifically, We engineer the conditional diffusion model to address the NILM task, where the total active power and embedded time tags are fed to the model as conditional input, and the appliance power waveform is generated step-by-step from Gaussian noise.
- We propose an encoding method for multi-scale temporal features that takes into account the regularity of power consumption behaviors.
- We implement and evaluate the proposed method on two public datasets, REDD and UKDALE. Empirical results demonstrate that DiffNILM outperforms previous models, as evidenced by both classification metrics and regression metrics.

## 2. Related Works

The overall framework of NILM was pioneered by Professor Hart in the 1980s, as documented in [6]. This approach was based on the notion that electric appliances exhibit unique features during state transition, which formed the basis for the event-based load monitoring method. However, Hart’s original approach only extracted steady-state features, which proved inadequate for appliances with multiple states and relatively low power consumption. To improve Hart’s algorithm, researchers discovered that repeatable transient profiles could be observed with high sampling rates, which allowed for the recognition of appliances’ transient signatures [7]. Various signal processing techniques, including Fourier Transform [8], Wavelet Transform [9], and Hilbert Transformation [10], were attempted to process transient power, current, and voltage signals.

To improve the identification accuracy, multiple electrical parameters were combined as input. The most prevailing method is the V-I trajectory, which maps current and voltage signals as a 2D image. Both steady-state and transient data can be utilized to generate V-I trajectories. For instance, Wang et al. [11] extracted the V-I trajectory based on steady-state data and developed an approach to quantify ten trajectory features. The research work in [12] utilized instantaneous voltage and current waveforms and proposed an algorithm that demonstrated high precision and strong robustness.

NILM based on traditional machine learning methods is mainly realized by the Hidden Markov Model (HMM) [13,14,15], Conditional Random Field (CRF) [16], and Support Vector Machine (SVM) [17]. These algorithms are supported by explicable mathematical principles, but their performances are often constrained by stringent assumptions (the characterization of load state transitions may not align with the actual operational features of various appliances), leading to limited accuracy and generalization abilities. Efforts have also been made to tackle the issue by framing it as a Combinatorial Optimization (CO) problem [18], but this method has proven to be computationally intractable, since it relies on enumeration.

With the significant progress of Deep Learning (DL), DL-based solutions have brought fresh insights to the practical advancement of artificial intelligence, which have been extensively adopted in various fields, including Computer Vision, Natural Language Processing, Signal Processing, etc. The application of DL-based techniques to energy disaggregation started with Kelly and Knottenbelt’s pioneering work in 2015 [19], where they introduced three deep neural network architectures to NILM, surpassing CO and diverse HMM-based algorithms in terms of both accuracy and generalization capability. Since then, DL-based methods have gradually dominated NILM research.

Recurrent Neural Networks (RNNs) are a type of deep learning architecture particularly well-suited for handling sequential data [20]. However, the vanishing gradient problem has posed a major challenge in the field. To address this issue, Long Short-Term Memory (LSTM) networks [21] have been commonly used in NILM. Convolutional Neural Networks (CNNs) have proven to be highly effective for image tasks and excel in sequential data analysis as well [22]. Zhang et al. [23] compared Seq2Point and Seq2Seq learning approaches using CNN-based mappings for training.

The models mentioned above have also been optimized to enhance computational efficiency since real-time load disaggregation is crucial for certain use cases, such as DSR and fault detection [24,25]. While accurate approaches have been proposed, there are also light-weight approaches to enable online computation, including a super-state hidden Markov model and a new variant of the Viterbi algorithm in an HMM-based framework for computationally efficient exact inference [26], as well as methods based on Gated Recurrent Unit (GRU), which reduce memory usage and computational complexity [27,28]. In addition, an experimental platform has been developed to realize real-time computation with a calculation time limit of one second [29].

In the past few years, Attention Mechanism has gained widespread popularity in handling sequential data processing tasks. The fundamental idea is to direct focus onto the most essential segment in the input sequence by assigning the highest weights to the most relevant parts. Capitalizing on the advantages of Attention Mechanism, Google introduced the Transformer architecture in 2017 [30], which allows parallel computation, as opposed to RNNs, and demonstrates a significantly superior capability to capture sequential features compared to CNNs. Building upon Transformer, the research work in [31,32] designed an architecture, based on Bidirectional Encoder Representations, from Transformers (BERT) for NILM, and proposed comprehensive loss functions that incorporate both regression and classification metrics.

NILM can also be regarded as a generation task aimed at creating synthetic waveforms for individual appliances, so the implementation of deep generative models, which are capable of modeling the underlying distribution of the power data, has been explored. A deep latent generative model for NILM, based on the Variational Recurrent Neural Network (VRNN), has been proposed, which performs sequence-to-many-sequence prediction [33]. The strong generational ability of the Variational Autoencoder (VAE) improves the formation of complex load profiles [34]. Conditional Generative Adversarial Network (cGAN) was used to avoid manually designing loss functions [35]. The work in [36] unified auto-encoder and GAN to realize the source separation of nonlinear power signals. Drawing inspiration from the favorable outcomes attained by several non-autoregressive generative models, the proposed study endeavors to employ the diffusion model, a more advanced approach, to the task of NILM.

## 3. Denoising Diffusion Probabilistic Models

With inspiration from non-equilibrium thermodynamics, the basic idea of DDPMs is to destroy the original data by gradually adding Gaussian noise, and then to learn to reconstruct the data through an inference process. The noising and denoising Markov chains are defined as the forward process and reverse process, respectively.

**Figure 1:**
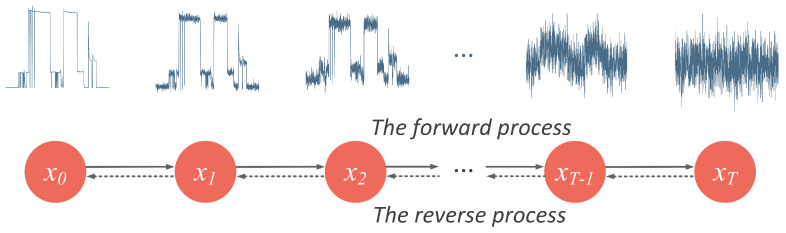
Illustration of the forward process and the reverse process in a NILM task. The pink arrows point out the process of forward diffusion, where a clean power pattern is gradually destroyed. The green arrows indicate the process of denoising inference, where the target waveform is recovered.

The step-by-step destruction and reconstruction of a power waveform in a diffusion model is illustrated in Figure 1. Random noise is successively added to $x_{0}$, a segment of clean power waveform of the target appliance, until the discernible features are completely lost. In reverse, we start from a random Gaussian noise $x_{T}$ and progressively remove extra noise to generate the target distribution. The original data $x_{0}$ and whitened latent variables $x_{1},x_{2},\ldots ,x_{T}$ share the same dimensionality.

### 3.1. The Forward Process

Diffusion models can be seen as latent variable models which create mappings to a hidden feature space, and this process is controlled by a predefined linear schedule $\beta_{1:T}=\left[\beta_{1},\beta_{2},\ldots ,\beta_{T}\right]$. According to the defining characteristic of the Markov chain, the distribution of $x_{t}$ at any arbitrary time step depends solely on its previous state $x_{t-1}$, so we add Gaussian noise to a $x_{t}$ by means of the following formula:

(1) $$x_{t}=\sqrt{a_{t}}x_{t-1}+\sqrt{1-\alpha_{t}}\varepsilon_{t}$$

where $\alpha_{t}=1-\beta_{t}$ and $\varepsilon_{t}∼N\left(0,I\right)$. The iterative formula of the forward process is given as:

(2) $$q\left(x_{t}|x_{t-1}\right)=N\left(x_{t};\sqrt{1-\beta_{t}}x_{t-1},\beta_{t}I\right)$$

In order to spare us from having to do step-by-step iteration, we derived the closed-form expression to directly calculate $x_{t}$ by $x_{0}$ using a reparameterization trick:

(3) $$q\left(x_{t}|x_{0}\right)=N\left(x_{t};\sqrt{{\bar{\alpha }}_{t}}x_{0},\left(1-{\bar{\alpha }}_{t}\right)I\right)$$

where ${\bar{\alpha }}_{t}=\prod_{i=1}^{t}\alpha_{i}$.

In many applications of the diffusion process, the parameters $\beta_{1:T}$ are often assigned small values following an increasing pattern. For instance, in [1], $\beta_{1:T}$ is defined as a linear function with values ranging from $10^{-4}$ to 0.02 over 1000 time steps. As *T* grows sufficiently large, ${\bar{\alpha }}_{t}$ converges to zero, and the resulting distribution of the latent variable $x_{T}$ approaches a standard normal distribution. The diffusion process ceases when the final distribution becomes sufficiently disordered to be considered an isotropic Gaussian distribution.

### 3.2. The Reverse Process

**Figure 2:**
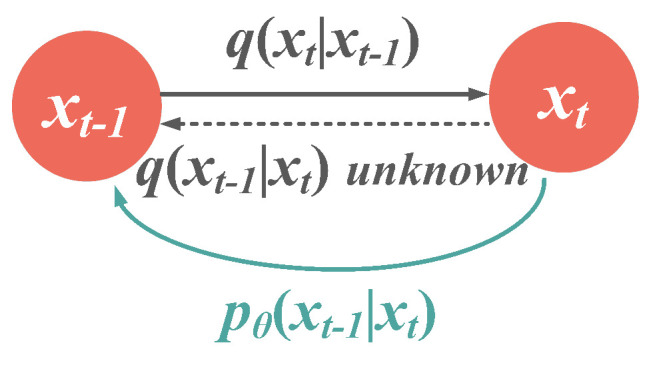
Illustration of transfer distributions between two diffusion steps. With the unsolvable $q\left(x_{t-1}|x_{t}\right)$, we approximate this reverse distribution with a parameterized model $p_{\theta }$.

The reverse process is where the desired output data is generated by tracing the Markov chain backward. Starting from $x_{T}$, if the distribution of any $x_{t-1}$ can be derived from the prior term $x_{t}$, the original distribution $x_{0}$ can be recovered from pure Gaussian noise. Unfortunately, the reverse transfer distribution $q\left(x_{t-1}|x_{t}\right)$ is not inferrable by simple mathematical derivation, so we used a deep learning model with parameter θ to estimate this reverse distribution, as depicted in Figure 2.

Conditioned on $x_{0}$, the reverse conditional probability can be derived on the basis of the Bayes Rule:

(4) $$\begin{array}{cc} q\left(x_{t-1}|x_{t},x_{0}\right) & =q\left(x_{t}|x_{t-1},x_{0}\right)\frac{q\left(x_{t-1}|x_{0}\right)}{q\left(x_{t}|x_{0}\right)}\\ \; & \propto \exp\left(-\frac{1}{2}\left(\frac{{\left(x_{t}-\sqrt{\alpha_{t}}x_{t-1}\right)}^{2}}{\beta_{t}}+\frac{{\left(x_{t-1}-\sqrt{\alpha_{t-1}}x_{0}\right)}^{2}}{1-{\bar{\alpha }}_{t-1}}-\frac{{\left(x_{t}-\sqrt{\alpha_{t}}x_{0}\right)}^{2}}{1-{\bar{\alpha }}_{t}}\right)\right)\\ \; & =\exp\left(-\frac{1}{2}\left(\left(\frac{\alpha_{t}}{\beta_{t}}+\frac{1}{1-{\bar{\alpha }}_{t-1}}\right)x_{t-1}^{2}-\left(\frac{2\sqrt{\alpha_{t}}}{\beta_{t}}x_{t}+\frac{2\sqrt{\alpha_{t}}}{1-{\bar{\alpha }}_{t}}x_{0}\right)x_{t-1}+C\right)\right) \end{array}$$

where *C* is a term not involving $x_{t-1}$. According to the probability density function of the normal distribution, the mean and variance of Equation (4) can be expressed as:

(5) $$\left\{\begin{array}{c} {\tilde{\beta }}_{t}=\frac{1-{\bar{\alpha }}_{t-1}}{1-{\bar{\alpha }}_{t}}\cdot \beta_{t}\\ {\tilde{\mu }}_{t}\left(x_{t},x_{0}\right)=\frac{\sqrt{\alpha_{t}}\left(1-{\bar{\alpha }}_{t-1}\right)}{1-{\bar{\alpha }}_{t}}x_{t}+\frac{\sqrt{{\bar{\alpha }}_{t-1}}\beta_{t}}{1-{\bar{\alpha }}_{t}}x_{0} \end{array}\right.$$

Then, we transform Equation (1) into the form of $x_{0}=\frac{1}{\sqrt{{\bar{a}}_{t}}}\left(x_{t}-\sqrt{1-{\bar{a}}_{t}}{\bar{\varepsilon }}_{t}\right)$ to replace the unknown $x_{0}$ in Equation (5), and derive the target mean that only depends on $x_{t}$:

(6) $${\tilde{\mu }}_{t}\left(x_{t}\right)=\frac{1}{\sqrt{\alpha_{t}}}\left(x_{t}-\frac{\beta_{t}}{\sqrt{1-{\bar{\alpha }}_{t}}}\varepsilon_{t}\right)$$

The above derivations reveal that the variance ${\tilde{\beta }}_{t}$ relies solely on the noise schedule and, thus, can be pre-computed. The parameter to be approximated ($\varepsilon_{t}$) exists in ${\tilde{\mu }}_{t}$, so we use a neural network to estimate the noise and, consequently, the mean.

### 3.3. Training a Diffusion Model

Diffusion models adopt the modeling method of noise prediction, where the neural networks take $x_{t}$ and time step *t* as input to estimate the noise $\varepsilon_{\theta }\left(x_{t},t\right)$. The goal of the training process is to narrow the gap between the actual noise and the predicted one by optimizing the negative log-likelihood using the variational lower bound. The loss term is parameterized as:

(7) $$\begin{array}{cc} L_{t}\left(\theta \right) & =E_{x_{0},t,\epsilon }\left[\frac{1}{2{∥\Sigma_{\theta }\left(x_{t},t\right)∥}_{2}^{2}}{∥{\tilde{\mu }}_{t}-\mu_{\theta }\left(x_{t},t\right)∥}_{2}^{2}\right]\\ \; & =E_{x_{0},t,\epsilon }\left[\frac{\beta_{t}^{2}}{2\alpha_{t}\left(1-{\bar{\alpha }}_{t}\right){∥\Sigma_{\theta }∥}_{2}^{2}}{∥\epsilon -\epsilon_{\theta }\left(\sqrt{{\bar{a}}_{t}}x_{0}+\sqrt{1-{\bar{a}}_{t}}\epsilon ,t\right)∥}^{2}\right] \end{array}$$

A few simplifications lead to more stable training:

(8) $$L_{\mathrm{simple}\;}\left(\theta \right)=E_{x_{0},t,\epsilon }\left[{∥\epsilon -\epsilon_{\theta }\left(\sqrt{{\bar{\alpha }}_{t}}x_{0}+\sqrt{1-{\bar{\alpha }}_{t}}\epsilon ,t\right)∥}^{2}\right]$$

## 4. Design

### 4.1. Conditional Diffusion Model as Appliance-Level Data Generator

One of the salient features of NILM, as a generation task, is that, instead of randomly generating power sequences that follow a certain distribution, the generation of each segment of appliance power waveform is conditioned on a segment of aggregated power waveform with the same length. However, the vanilla diffusion model was originally designed for unconditional image generation, which necessitates adaptive modifications to tailor it to the requirements of the NILM task.

Conditional diffusion models have been well-studied in other sequence modeling tasks. For instance, in machine translation the model conditions on the source sentences, and in speech synthesis the model conditions on the mel-spectrogram. The general goal of such algorithms is to model the probability density of $p_{\theta }\left(x_{0}|x_{d}\right)$, where $x_{d}$ contains conditioning features relevant to $x_{0}$. For diffusion models, the conditional distribution can be written as:

(9) $$p_{\theta }\left(x_{0}|x_{d}\right):=\int p_{\theta }\left(x_{0:N}|x_{d}\right)dx_{1:N}$$

**Figure 3:**
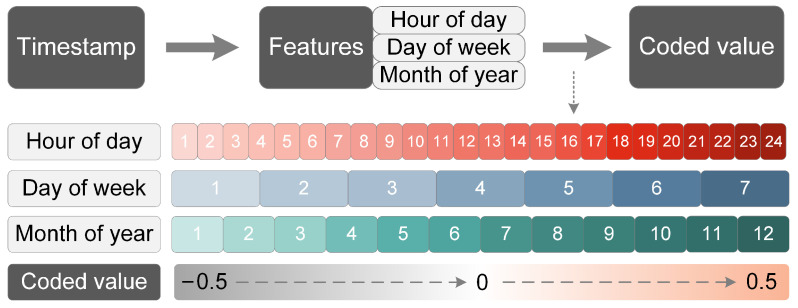
Illustration of temporal data encoding.

The proposed model takes two conditional inputs: the total power and the encoded temporal features. Traditional NILM algorithms only detect the states of appliances based on the aggregated power sequence, disregarding the regularity and periodicity in the energy consumption patterns of users (for instance, dishwashers are generally used after dinner, and refrigerators operate more frequently during summer). In this study, we present an encoding technique that integrates multi-scale temporal information as supplementary knowledge for energy disaggregation, with reference to the global timestamp representation introduced in [37]. As illustrated in Figure 3, we extract three features from each time tag: hour of day, day of week and month of year, and then linearly encode these three features into values within the interval of [−0.5, 0.5], respectively.

Moreover, the continuous noise level is adopted in this paper, as opposed to discrete noise level, where we sample t∼Uniform({1,2,…,T}) and reach for the corresponding $\alpha_{t}$ in the predefined linear schedule. The proposed diffusion model conditions on the continuous noise level $\sqrt{\bar{\alpha }}$ instead of time step *t* and $\sqrt{\bar{\alpha }}$ is randomly chosen between two adjacent discrete noise levels:

(10) $$\sqrt{\bar{\alpha }}∼\mathrm{Uniform}\left(\sqrt{{\bar{\alpha }}_{t}},\sqrt{{\bar{\alpha }}_{t-1}}\right)$$

**Figure 4:**
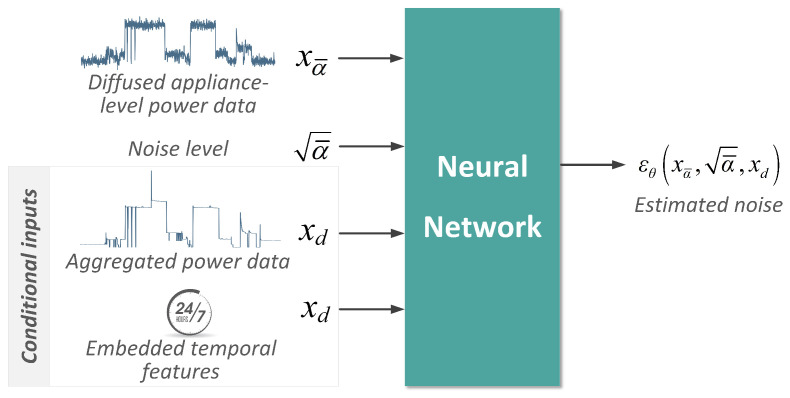
Conditional diffusion model for NILM task. The neural network takes diffused data $x_{\bar{\alpha }}$, noise level $\sqrt{\bar{\alpha }}$ and conditional data $x_{aggre}$ and $x_{times}$ as inputs to estimate the corresponding noise $\varepsilon_{\theta }$.

For our task, as depicted in Figure 4, the neural network takes three inputs: the noisy appliance-level power data $x_{t}$, the corresponding noise level $\sqrt{\bar{a}}$, the conditional aggregated power data $x_{aggre}$ and embedded time tags $x_{time}$, and outputs the approximated noise $\varepsilon_{\theta }\left(x_{t},\sqrt{\bar{a}},x_{aggre},x_{time}\right)$.

### 4.2. Network Architecture

This section details the implementation of a neural network for noise prediction, with an architecture inspired by NU-Wave [38] and DiffWave [4], which are two diffusion-based neural vocoders.

**Figure 5:**
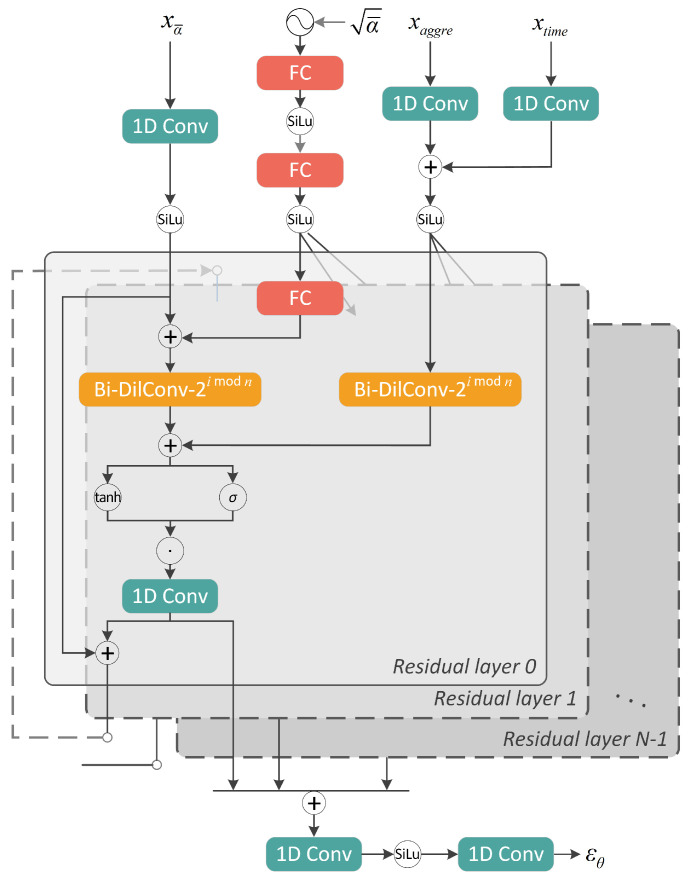
The neural network architecture for noise prediction.

As revealed in Figure 5, 1D convolutional layers were used to increase the number of channels of the input sequences $x_{\bar{\alpha }}$, $x_{aggre}$ and $x_{time}$ to *C*, and the Sigmoid Linear Unit (SiLU) activation was adopted:

(11) SiLu(x)=x∗Sigmoid(x)

Similar to the positional embedding method, proposed in Transformer [30], the sinusoidal encoding formula is applied to embed the noise level $\sqrt{\bar{\alpha }}$:

(12) $$Embed\left(\sqrt{\bar{\alpha }}\right)=\left[\sin\left(10^{-\frac{\left[0:63\right]}{16}}\times 50,000\times \sqrt{\bar{\alpha }}\right),\cos\left(10^{-\frac{\left[0:63\right]}{16}}\times 50,000\times \sqrt{\bar{\alpha }}\right)\right]$$

Then we use two shared SiLu-activated Fully Connected (FC) layers and one residual-layer-specific FC layer to project the encoded noise level to a *C*-dimensional vector, and add it to the convoluted $x_{\bar{\alpha }}$ as a bias term.

The main body of the model consists of *N* conformably-structured layers connected in residual manner to enable the direct delivery of input information to the final layers. In each residual layer, we used Bi-directional Dilated Convolution (Bi-DilConv) to deal with the inputs for an exponential growth in the receptive field, and in the *i*-th residual layer, the spacing between the kernel points was set to $2^{i\;\mathrm{mod}\;n}$. Gated Units (GU) are applied to activate the summation of the processed noisy signals and conditional signals. Then, the convoluted vector is split in two and passed on as residual output and skip output, respectively. Finally, we sum all the skip connections and use two convolutional layers to gain the noise vector $\varepsilon_{\theta }$ in the same shape as $x_{\bar{\alpha }}$ and $x_{aggre}$.

### 4.3. Training and Sampling Procedures

The training and sampling procedures of the diffusion model are shown in Algorithms 1 and 2. In the training procedure, after extracting data from the dataset, we sample an iteration index *t* and obtain a corresponding continuous noise level $\sqrt{\bar{\alpha }}$ to determine the extent of whitening applied to the original waveform. As mentioned in Section 3.3, the deep learning model updates its parameters with the purpose of minimizing the distance between the sampled noise ε and the predicted noise $\varepsilon_{\theta }$. Instead of using common loss functions, such as MSE and $L_{1}$ norm, we found that log-norm improves convergence speed and leads to improved empirical outcomes:

(13) $$\log{∥\varepsilon -\varepsilon_{\theta }\left(x_{\bar{\alpha }},\sqrt{\bar{\alpha }},x_{d}\right)∥}_{1}$$

In the sampling algorithm, we adopted a fast sampling method where much fewer inference steps are used. Instead of traversing the reverse process step by step with t=T,T−1,…,1, we define an inference schedule with only $T_{infer}$ noise levels ($T_{infer}<<T$). The test results demonstrated that the fast sampling trick greatly accelerated the inference procedure without degrading generational quality. In each inference step, we calculate the predicted variance ${\tilde{\beta }}_{t}$ and mean $\mu_{\theta }\left(x_{\bar{\alpha }},\sqrt{\bar{\alpha }},x_{d}\right)$ to estimate the previous term.
**Algorithm 1:** Training.
  1:    **repeat**
  2:      $x_{0}∼q\left(x_{0}\right)$
  3:      t∼Uniform({1,2,…,T})
  4:      $\sqrt{\bar{\alpha }}∼\mathrm{Uniform}\left(\sqrt{{\bar{\alpha }}_{t}},\sqrt{{\bar{\alpha }}_{t-1}}\right)$
  5:      ε∼N(0,I)
  6:       Take gradient descent step on $\nabla_{\theta }\log{∥\varepsilon -\varepsilon_{\theta }\left(x_{\bar{\alpha }},\sqrt{\bar{\alpha }},x_{d}\right)∥}_{1}$
  7:    **until** converged


**Algorithm 2:** Sampling.
  1:    $x_{T}∼N\left(0,I\right)$
  2:    **for** $t=T_{infer},T_{infer}-1,\ldots ,1$ **do**
  3:      z∼N(0,I)
  4:      ${\tilde{\beta }}_{t}=\sqrt{\frac{1-{\bar{\alpha }}_{t-1}}{1-{\bar{\alpha }}_{t}}\beta_{t}}$
  5:      $\mu_{\theta }\left(x_{\bar{\alpha }},\sqrt{\bar{\alpha }},x_{d}\right)=\frac{1}{\sqrt{\alpha_{t}}}\left(x_{t}-\frac{\beta_{t}}{\sqrt{1-{\bar{\alpha }}_{t}}}\varepsilon_{\theta }\left(x_{\bar{\alpha }},\sqrt{\bar{\alpha }},x_{d}\right)\right)$
  6:      $x_{t-1}=\mu_{\theta }\left(x_{t},t\right)+{\tilde{\beta }}_{t}z$
  7:    **end for**
  8:    **return**
$x_{0}$


## 5. Experiments

**Figure 6:**
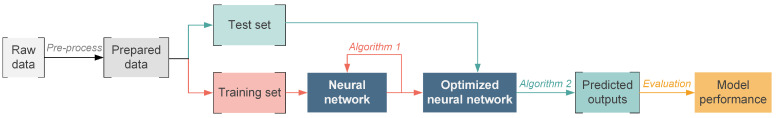
The workflow of the experiment conducted.

We carried out an experiment to test the proposed model. The workflow, as illustrated in Figure 6, involved pre-processing data, splitting the dataset, training a neural network using the training set, and evaluating its performance on the testing set.

### 5.1. Dataset

This study employed low-frequency active power data from the REDD and UKDALE datasets to train and test the proposed model. REDD is the most widely-used dataset in the domain of NILM, comprising the mains and submeter power data of six residential homes in the United States, recorded over a period of approximately four months. The UKDALE dataset, on the other hand, was published by Imperial College London, in 2014, and contains power consumption information from House 1 collected for up to three years, while the data for the other four houses were recorded for several months.

We pre-processed the original power data according to the following procedure:

Step 1: Merge the data of split-phase mains meter. Two-phase power supply is commonly-used in North American households, so, for REDD, we calculated the sum of each mains meter to obtain the actual aggregated power data.
Step 2: Resample the power data at a fixed interval of 6 s.
Step 3: Fill data gaps shorter than 3 min by forward-filling, and fill those longer than 3 min with zeros.
Step 4: Attach status labels to the datasets. An appliance is classified as being in an ‘on’ state at a particular time point and assigned a status label of 1, provided that its power consumption falls within the acceptable ‘on’ power range and its operation time exceeds the minimum duration specified in Table 1. Otherwise, a status label of 0 is assigned.
Step 5: Standardize the power data according to Formula (14) to enhance the accuracy of the model and convergence speed.

(14) $$x^{*}=\frac{x-\mu }{\sigma }$$

Following the pre-processing of the power data, overlapping sliding windows were utilized to extract sequences of processable length.

### 5.2. Evaluation Metrics

The selection of suitable metrics is important in appraising the algorithm’s performance. As NILM can be formulated as either a binary classification problem (to detect the on/off states of the target appliance) or a regression problem (to estimate the numeric value of power consumption), the evaluation incorporated both classification and regression metrics to ensure a comprehensive assessment.

#### 5.2.1. Classification Metrics

We used classification metrics in Equation (15) to evaluate the ability of the algorithm to identify the on/off states, where TP, FP and FN, respectively, represent the number of TP (True Positive), FP (False Positive) and TN (True Negative) results, and *P* and *N*, respectively, represent the number of points where the appliance is switched on and off in ground truth.

(15) $$\begin{array}{c} accuracy=\frac{TP+TN}{P+N}\\ recall=\frac{TP}{TP+FN}\\ precision=\frac{TP}{TP+FP}\\ F\text{-}score=\left(1+\beta^{2}\right)\times \frac{precision\times recall}{\beta^{2}\times precision+recall}\\ F1\text{-}score=2\times \frac{precision\times recall}{precision+recall} \end{array}$$

While accuracy is an intuitive classification metric, its applicability is restricted in datasets that are unbalanced, where the ‘on’ states of appliances constitute a small fraction of the entire sequence. In such cases, the F-score index serves as an effective approach to address the imbalance issue. The F-score comprehensively incorporates both precision and recall, and varying weights can be assigned to them by adjusting the β value, thereby enabling an evaluation of the quality of NILM algorithms under diverse application scenarios. Given that precision and recall are usually deemed equally important, the value of β was set to 1, and F-score was calculated as the harmonic average of the two, termed as F1-score.

#### 5.2.2. Regression Metrics

To evaluate the performance of the model to reconstruct the power profiles of the target appliance, two commonly-used regression metrics, Mean Absolute Error (*MAE*) and Mean Relative Error (*MRE*), were adopted:

(16) $$\begin{array}{c} \begin{array}{c} MAE=\frac{1}{T}\sum_{t=1}^{T}|{\hat{x}}_{t}-x_{t}|\\ MRE=\frac{1}{T}\sum_{t=1}^{T}\frac{{\hat{x}}_{t}-x_{t}}{\max({\hat{x}}_{t},x_{t})} \end{array} \end{array}$$

where ${\hat{x}}_{t}$ and $x_{t}$, respectively, represent the appliance’s estimated and actual power at time *t*, and *T* is the total number of points in the sequence.

### 5.3. Implementation Details

The NILM project was conducted on a 64-bit computer equipped with Intel(R) CoreTM i7-12700 CPU@ 3.61 GHz, 32 GB memory, and NVIDIA GeForce RTX 3080Ti. The Pytorch framework was employed to train and test the diffusion model.

During the training phase, the model was trained until convergence at a learning rate of $3\times 10^{-5}$. To accelerate gradient descent, the Adam optimizer was utilized, where the hyperparameters $\beta_{1}$ and $\beta_{2}$ were set to 0.5 and 0.999, respectively.

The hyperparameters of the diffusion model are shown in Table 2.

### 5.4. Results

**Figure 7:**
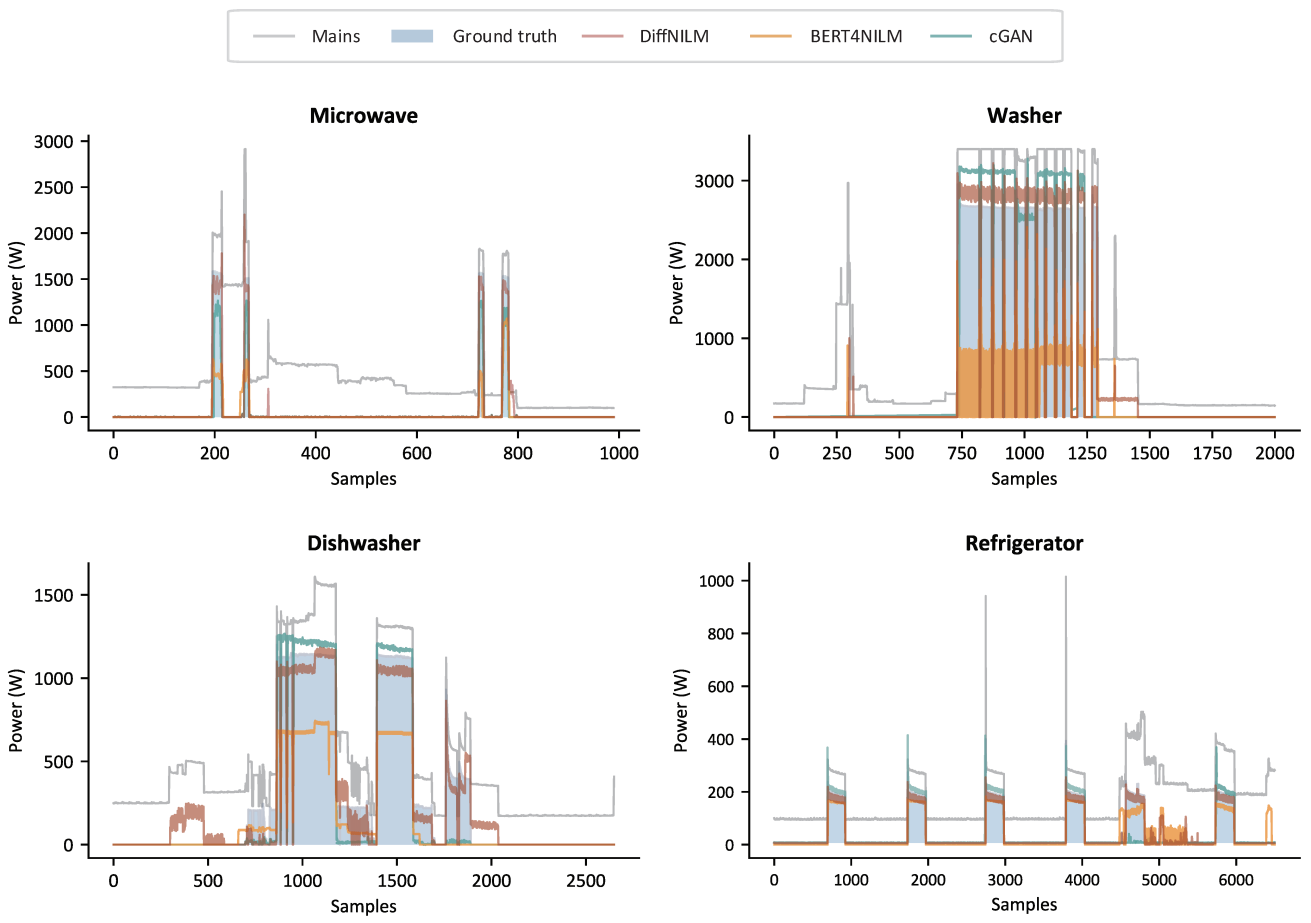
Sample outputs of microwave, Washer, dishwasher and refrigerator on REDD.

**Figure 8:**
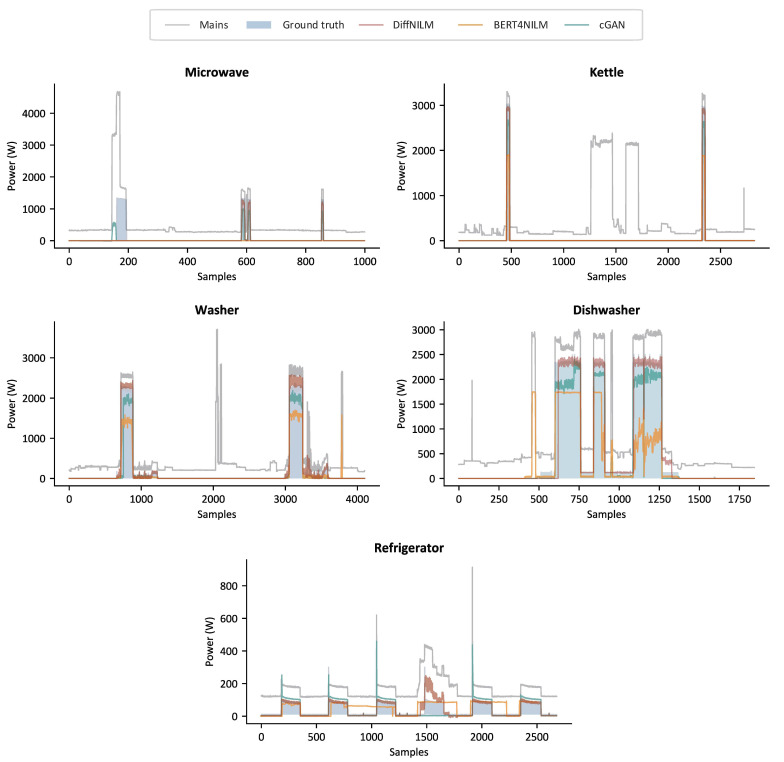
Sample outputs of microwave, kettle, Washer, dishwasher and refrigerator on UKDALE.

DiffNILM was evaluated against four state-of-the-art NILM models, including the bi-directional LSTM [21], CNN [23], BERT4NILM [31] and cGAN [35]. The objectivity of the comparative experiments was ensured by adopting the same data processing method, and all the baseline models were trained to convergence. The performance indicators of the five disaggregation models on REDD and UKDALE datasets are shown in Table 3 and Table 4. Output sample curves generated by DiffNILM, BERT4NILM, and cGAN models are displayed in Figure 7 and Figure 8, where two relatively underperforming methods were excluded to avoid clutter.

For starters, we examined the performance of DiffNILM on microwaves and kettles, which are characterized by infrequent usage and relatively short running periods. The results from the tables indicate that the proposed algorithm outperformed other methods on several indicators, particularly the MAE and MRE. The output signals further reveal that the model effectively captured most of the activations and the predicted power values aligned well with the ground truth values. However, a few exceptional cases were identified where the power signatures were not exactly typical, notably in the first activation of the microwave, depicted in Figure 8, which exhibited a longer turn-on time than other instances, and was subject to relatively strong interference from background noise.

Washers and dishwashers are a type of household appliances that exhibit infrequent use but extended operation per use. The consumption patterns are intricate, due to the frequent start-and-stop events and mode switching during operation. In the REDD dataset, Washers maintained a constant power level during the ‘on’ mode and the waveforms were effectively rebuilt by DiffNILM, despite the slightly elevated power values. Washers in the UK have operating patterns that are distinct from their US counterparts, with evident power oscillations, which the proposed algorithm effectively reconstructed. Dishwashers present more complex operational characteristics with multiple modes, such as pre-rinse, steam wash and dry, which require a more advanced model generation capacity. Although DiffNILM’s output in low power consumption mode was not entirely consistent with the ground truth signal, it exhibited good overall power estimation performance.

The refrigerator operates based on automatic temperature regulation requirements, with frequent start and stop events and prominent periodicity. Based on the evaluation metrics and sample waveforms, DiffNILM exhibited satisfactory performance in disaggregating the refrigerator load. The algorithm could accurately detect each activation event, and the power prediction accuracy was only compromised when there was significant background power interference.

Through horizontal comparison of the results on the two datasets, it is interesting to notice that the metrics of the two generative models exhibited more enhanced performance on UKDALE than REDD. A plausible reason for this outcome is that deep generative models typically necessitate larger amounts of training data. Specifically, the smaller REDD dataset might fail to meet the data requirements of cGAN and DiffNILM, which, in turn, would hinder their performances on this dataset. In contrast, the larger UKDALE dataset facilitated better performance, reflected in the significant improvement of the metrics of the generative models.

Overall, the proposed algorithm outperformed the baseline models on most metrics and yielded better results than the previous methods concerning the mean values of the four metrics on both datasets. Meanwhile, DiffNILM demonstrated a satisfactory fitting effect on the consumption signals of various electrical appliances, and was capable of handling complex load patterns. Nonetheless, due to the unique nature of ’diffusion’, the predicted power curve was not always smooth. Additionally, in cases where the background power was complex, the disaggregated curve might experience distortion following the total power, although the impact remained within an acceptable range.

## 6. Conclusions

In this paper, we introduce DiffNILM, a novel framework for energy disaggregation that utilizes the diffusion probabilistic model. The key innovation of our approach is the conditional diffusion model which takes both the total active power and embedded time tags as inputs and generates the appliance power waveforms. Additionally, we propose an encoding method for multi-scale temporal features which captures the periodicity of power consumption behaviors. The proposed method was applied and assessed on two open-access datasets, REDD and UKDALE. Averaging across all appliances, DiffNILM displayed an improvement in all four metrics on both datasets. The results also highlight the potential of the proposed DiffNILM algorithm in reconstructing complex load patterns, despite the fact that DiffNILM exhibits certain issues, such as generating power waveforms that are not sufficiently smooth and may experience distortion.

Meanwhile, we would like to clarify that the algorithm was developed with accuracy as the primary objective, and we did not explicitly consider the computational burden of the proposed implementation. Going forward, we are committed to developing a light-weight version of the algorithm that balances both accuracy and computational efficiency. This will enable the approach to be deployed in real-world settings with limited computational resources.

Furthermore, when analyzing the results, the significance of dataset size in achieving optimal performance was noted. However, acquiring large-scale appliance-level data through field sampling in numerous households can be a formidable task. In forthcoming research, we aim to explore a method of synthesizing appliance power signatures as a means of augmenting the existing NILM datasets, which can also be realized with diffusion models.

## Figures and Tables

**Table 1:** Basic parameter settings of appliances.

Appliance	Reasonable ‘on’ Power Range (W)	Minimum Duration of Operation (s)
Microwave	200∼1800	12
Washer	40∼3500	1800
Dish washer	50∼1200	1800
Refrigerator	50∼400	60

**Table 2:** Hyperparameters of the model.

Symbol	Description	Value
*L*	Length of the input and output power sequences	480
*T*	Maximum diffusion step	1000
$\beta_{1:T}$	Noise schedule	$\mathrm{Linear}\left(1\times 10^{-6},0.006,1000\right)$
$T_{infer}$	Inference step	8
$\beta_{1:T_{infer}}$	Inference noise schedule	$1\times 10^{-6},2\times 10^{-6},1\times 10^{-5},1\times 10^{-4},1\times 10^{-3},1\times 10^{-2},1\times 10^{-1},9\times 10^{-1}$
*N*	Number of residual layers	30
*C*	Number of residual channels	128
*n*	Length of the dilation cycle	10

**Table 3:** Model performances on REDD.

Appliance	Model	Accuracy ↑	F1-Score ↑	MAE ↓	MRE ↓
	Bi-LSTM	0.989	**0.604**	17.39	0.058
	CNN	0.986	0.378	18.59	0.060
Microwave	BERT4NILM	0.989	0.476	17.58	0.057
	cGAN	0.989	0.415	18.15	0.058
	DiffNILM	**0.989**	0.430	**17.13**	**0.057**
	Bi-LSTM	0.989	0.125	35.73	0.020
	CNN	0.970	0.274	36.12	0.042
Washer	BERT4NILM	0.991	0.559	34.96	0.022
	cGAN	0.990	0.478	29.67	0.025
	DiffNILM	**0.988**	**0.569**	**26.44**	**0.019**
	Bi-LSTM	0.956	0.421	25.25	0.056
	CNN	0.953	0.298	25.29	0.053
Dish washer	BERT4NILM	0.969	0.523	20.49	0.039
	cGAN	0.951	0.295	24.80	0.055
	DiffNILM	**0.971**	**0.593**	**18.16**	**0.037**
	Bi-LSTM	0.789	0.709	44.82	0.841
	CNN	0.796	0.689	35.69	0.822
Refrigerator	BERT4NILM	0.841	0.756	**32.35**	**0.806**
	cGAN	0.811	0.732	33.83	0.820
	DiffNILM	**0.868**	**0.794**	33.58	0.808
	Bi-LSTM	0.931	0.465	30.80	0.244
	CNN	0.926	0.410	28.92	0.244
Average	BERT4NILM	0.948	0.579	26.35	0.231
	cGAN	0.935	0.498	26.70	0.240
	DiffNILM	**0.954**	**0.597**	**23.70**	**0.230**

↑ indicates that a higher value of the metric is better, while ↓ indicates that a lower value of the metric is better. The bold item in each column represents the optimal index for that particular metric in all the models.

**Table 4:** Model performances on UKDALE.

Appliance	Model	Accuracy ↑	F1-Score ↑	MAE ↓	MRE ↓
	Bi-LSTM	0.995	0.060	6.55	0.014
	CNN	0.995	0.341	6.36	0.014
Microwave	BERT4NILM	0.995	0.014	6.57	0.014
	cGAN	0.996	0.474	5.98	0.012
	DiffNILM	**0.996**	**0.501**	**4.54**	**0.012**
	Bi-LSTM	0.938	0.150	15.66	0.067
	CNN	0.913	0.173	11.90	0.094
Washer	BERT4NILM	0.966	0.325	6.98	0.040
	cGAN	0.959	0.376	10.84	0.062
	DiffNILM	**0.986**	**0.390**	**5.74**	**0.058**
	Bi-LSTM	0.976	0.605	36.36	0.033
	CNN	0.947	0.560	25.45	0.069
Dish washer	BERT4NILM	0.966	0.667	16.18	0.049
	cGAN	0.961	**0.646**	**13.89**	0.042
	DiffNILM	**0.980**	0.662	19.58	**0.030**
	Bi-LSTM	0.573	0.174	43.74	0.956
	CNN	0.772	0.718	29.29	0.758
Refrigerator	BERT4NILM	0.813	0.766	25.47	0.732
	cGAN	0.818	0.801	25.11	0.730
	DiffNILM	**0.857**	**0.816**	**22.82**	**0.699**
	Bi-LSTM	0.994	0.531	21.26	0.007
	CNN	0.997	0.850	9.64	0.003
Kettle	BERT4NILM	0.998	0.907	6.82	0.002
	cGAN	0.998	0.911	7.09	0.002
	DiffNILM	**0.999**	**0.918**	**4.59**	**0.002**
	Bi-LSTM	0.875	0.229	21.80	0.261
	CNN	0.919	0.521	14.28	0.261
Average	BERT4NILM	0.943	0.503	11.47	0.194
	cGAN	0.946	0.642	14.58	0.170
	DiffNILM	**0.964**	**0.657**	**11.45**	**0.164**

↑ indicates that a higher value of the metric is better, while ↓ indicates that a lower value of the metric is better. The bold item in each column represents the optimal index for that particular metric in all the models.

## Data Availability

To access the public dataset used in this paper, please follow the links provided: http://redd.csail.mit.edu/, accessed on 1 March 2023, for REDD and https://jack-kelly.com/data/forUKDALE, accessed on 1 March 2023.

## References

[1] Ho J., Jain A., Abbeel P. (2020). Denoising diffusion probabilistic models. Adv. Neural Inf. Process. Syst..

[2] Sehwag V., Hazirbas C., Gordo A., Ozgenel F., Canton C. Generating High Fidelity Data from Low-density Regions using Diffusion Models. Proceedings of the IEEE/CVF Conference on Computer Vision and Pattern Recognition.

[3] Wolleb J., Sandkühler R., Bieder F., Valmaggia P., Cattin P.C. (2021). Diffusion Models for Implicit Image Segmentation Ensembles. arXiv.

[4] Kong Z., Ping W., Huang J., Zhao K., Catanzaro B. (2020). Diffwave: A versatile diffusion model for audio synthesis. arXiv.

[5] Luo S., Hu W. Diffusion probabilistic models for 3d point cloud generation. Proceedings of the IEEE/CVF Conference on Computer Vision and Pattern Recognition.

[6] Hart G.W. (1992). Nonintrusive appliance load monitoring. Proc. IEEE.

[7] Mukaroh A., Le T.T.H., Kim H. (2020). Background load denoising across complex load based on generative adversarial network to enhance load identification. Sensors.

[8] Kang H., Kim H. (2020). Household appliance classification using lower odd-numbered harmonics and the bagging decision tree. IEEE Access.

[9] Gillis J.M., Alshareef S.M., Morsi W.G. (2015). Nonintrusive load monitoring using wavelet design and machine learning. IEEE Trans. Smart Grid.

[10] Heo S., Kim H. (2021). Toward load identification based on the Hilbert transform and sequence to sequence long short-term memory. IEEE Trans. Smart Grid.

[11] Wang A.L., Chen B.X., Wang C.G., Hua D. (2018). Non-intrusive load monitoring algorithm based on features of V–I trajectory. Electr. Power Syst. Res..

[12] Hassan T., Javed F., Arshad N. (2013). An empirical investigation of VI trajectory based load signatures for non-intrusive load monitoring. IEEE Trans. Smart Grid.

[13] Kolter J.Z., Johnson M.J. REDD: A public data set for energy disaggregation research. Proceedings of the Workshop on Data Mining Applications in Sustainability (SIGKDD).

[14] Kim H., Marwah M., Arlitt M., Lyon G., Han J. Unsupervised disaggregation of low frequency power measurements. Proceedings of the 2011 SIAM International Conference on Data Mining.

[15] Kolter J.Z., Jaakkola T. Approximate inference in additive factorial hmms with application to energy disaggregation. Proceedings of the Artificial Intelligence and Statistics.

[16] Mao Y., Dong K., Zhao J. Non-intrusive load decomposition technology based on CRF model. Proceedings of the 2021 IEEE Sustainable Power and Energy Conference (iSPEC).

[17] Gong F., Han N., Zhou Y., Chen S., Li D., Tian S. A svm optimized by particle swarm optimization approach to load disaggregation in non-intrusive load monitoring in smart homes. Proceedings of the 2019 IEEE 3rd Conference on Energy Internet and Energy System Integration (EI2).

[18] Piga D., Cominola A., Giuliani M., Castelletti A., Rizzoli A.E. (2015). Sparse optimization for automated energy end use disaggregation. IEEE Trans. Control Syst. Technol..

[19] Kelly J., Knottenbelt W. Neural nilm: Deep neural networks applied to energy disaggregation. Proceedings of the 2nd ACM International Conference on Embedded Systems for Energy-Efficient Built Environments.

[20] Elman J.L. (1990). Finding structure in time. Cogn. Sci..

[21] Xia M., Wang K., Song W., Chen C., Li Y. (2020). Non-intrusive load disaggregation based on composite deep long short-term memory network. Expert Syst. Appl..

[22] LeCun Y., Bengio Y. (1995). Convolutional networks for images, speech, and time series. The Handbook of Brain Theory and Neural Networks.

[23] Zhang C., Zhong M., Wang Z., Goddard N., Sutton C. Sequence-to-point learning with neural networks for non-intrusive load monitoring. Proceedings of the AAAI Conference on Artificial Intelligence.

[24] He D., Lin W., Liu N., Harley R.G., Habetler T.G. (2013). Incorporating non-intrusive load monitoring into building level demand response. IEEE Trans. Smart Grid.

[25] Athanasiadis C.L., Papadopoulos T.A., Doukas D.I. (2021). Real-time non-intrusive load monitoring: A light-weight and scalable approach. Energy Build..

[26] Makonin S., Popowich F., Bajić I.V., Gill B., Bartram L. (2015). Exploiting HMM sparsity to perform online real-time nonintrusive load monitoring. IEEE Trans. Smart Grid.

[27] Rafiq H., Zhang H., Li H., Ochani M.K. Regularized LSTM based deep learning model: First step towards real-time non-intrusive load monitoring. Proceedings of the 2018 IEEE International Conference on Smart Energy Grid Engineering (SEGE).

[28] Krystalakos O., Nalmpantis C., Vrakas D. Sliding window approach for online energy disaggregation using artificial neural networks. Proceedings of the 10th Hellenic Conference on Artificial Intelligence.

[29] Hu M., Tao S., Fan H., Li X., Sun Y., Sun J. (2021). Non-intrusive load monitoring for residential appliances with ultra-sparse sample and real-time computation. Sensors.

[30] Vaswani A., Shazeer N., Parmar N., Uszkoreit J., Jones L., Gomez A.N., Kaiser Ł., Polosukhin I. (2017). Attention is all you need. arXiv.

[31] Yue Z., Witzig C.R., Jorde D., Jacobsen H.A. Bert4nilm: A bidirectional transformer model for non-intrusive load monitoring. Proceedings of the 5th International Workshop on Non-Intrusive Load Monitoring.

[32] Sykiotis S., Kaselimi M., Doulamis A., Doulamis N. (2022). Electricity: An efficient transformer for non-intrusive load monitoring. Sensors.

[33] Bejarano G., DeFazio D., Ramesh A. Deep latent generative models for energy disaggregation. Proceedings of the AAAI Conference on Artificial Intelligence.

[34] Langevin A., Carbonneau M.A., Cheriet M., Gagnon G. (2022). Energy disaggregation using variational autoencoders. Energy Build..

[35] Pan Y., Liu K., Shen Z., Cai X., Jia Z. Sequence-to-subsequence learning with conditional gan for power disaggregation. Proceedings of the ICASSP 2020—2020 IEEE International Conference on Acoustics, Speech and Signal Processing (ICASSP).

[36] Kaselimi M., Doulamis N., Voulodimos A., Doulamis A., Protopapadakis E. (2020). EnerGAN++: A generative adversarial gated recurrent network for robust energy disaggregation. IEEE Open J. Signal Process..

[37] Zhou H., Zhang S., Peng J., Zhang S., Li J., Xiong H., Zhang W. Informer: Beyond efficient transformer for long sequence time-series forecasting. Proceedings of the AAAI Conference on Artificial Intelligence.

[38] Lee J., Han S. (2021). Nu-wave: A diffusion probabilistic model for neural audio upsampling. arXiv.

